# Mediastinal Lymph Node Dissection versus Mediastinal Lymph Node Sampling for Early Stage Non-Small Cell Lung Cancer: A Systematic Review and Meta-Analysis

**DOI:** 10.1371/journal.pone.0109979

**Published:** 2014-10-08

**Authors:** Xiongfeng Huang, Jianmin Wang, Qiao Chen, Jielin Jiang

**Affiliations:** Jiangxi University of Traditional Chinese Medicine, Nanchang, China; UT MD Anderson Cancer Center, United States of America

## Abstract

**Objective:**

This systematic review and meta-analysis aimed to evaluate the overall survival, local recurrence, distant metastasis, and complications of mediastinal lymph node dissection (MLND) versus mediastinal lymph node sampling (MLNS) in stage I–IIIA non-small cell lung cancer (NSCLC) patients.

**Methods:**

A systematic search of published literature was conducted using the main databases (MEDLINE, PubMed, EMBASE, and Cochrane databases) to identify relevant randomized controlled trials that compared MLND vs. MLNS in NSCLC patients. Methodological quality of included randomized controlled trials was assessed according to the criteria from the Cochrane Handbook for Systematic Review of Interventions (Version 5.1.0). Meta-analysis was performed using The Cochrane Collaboration’s Review Manager 5.3. The results of the meta-analysis were expressed as hazard ratio (HR) or risk ratio (RR), with their corresponding 95% confidence interval (CI).

**Results:**

We included results reported from six randomized controlled trials, with a total of 1,791 patients included in the primary meta-analysis. Compared to MLNS in NSCLC patients, there was no statistically significant difference in MLND on overall survival (HR = 0.77, 95% CI 0.55 to 1.08; *P* = 0.13). In addition, the results indicated that local recurrence rate (RR = 0.93, 95% CI 0.68 to 1.28; *P* = 0.67), distant metastasis rate (RR = 0.88, 95% CI 0.74 to 1.04; *P* = 0.15), and total complications rate (RR = 1.10, 95% CI 0.67 to 1.79; *P* = 0.72) were similar, no significant difference found between the two groups.

**Conclusions:**

Results for overall survival, local recurrence rate, and distant metastasis rate were similar between MLND and MLNS in early stage NSCLC patients. There was no evidence that MLND increased complications compared with MLNS. Whether or not MLND is superior to MLNS for stage II–IIIA remains to be determined.

## Introduction

Lung cancer is a malignant lung tumor in which the cells of lung tissues grow uncontrollably. Worldwide, lung cancer is the most common cause of cancer-related death in both men and women. In 2014, statistics from the American Cancer Society estimated that there will be about 224,210 new cases of lung cancer in the U.S. and about 159,260 people will die due to this disease [1]. There are two major types of lung cancer: non-small cell lung cancer (NSCLC) and small cell lung cancer (SCLC). The most common form of the disease is NSCLC, which accounts for approximately 85% of lung cancers [2], [3].

NSCLC may be curable by surgical resection, but the extent of lymph node removal required and the impact of mediastinal node removal remains controversial [4]–[6]. While it is commonly accepted that surgical staging of mediastinal lymph node dissection (MLND) is important, the therapeutic efficacy of MLND is still under debate, whereas MLND requires a more extensive mediastinal dissection than mediastinal lymph node sampling (MLNS) and may lead to more complications [7].

A previous meta-analysis has shown that MLND improves long term survival in stage I–IIIA NSCLC patients [8], [9]. However, in the American College of Surgery Oncology Group (ACOSOG) Z0030 trial, which is a multicenter prospective randomized trial, the authors concluded that MLND does not improve survival in patients with early stage NSCLC, but the results are not generalizable to patients staged radiographically or those with higher stage tumors [10].

Clearly, newer systematic review and meta-analyses are required to resolve these differences, and definitive analyses can provide stronger rationales for the choice of a specific therapy. For these reasons, we performed a meta-analysis of pooled data from existing randomized controlled trials (RCTs) to evaluate the efficacy and safety of the MLND vs. MLNS in early stage NSCLC patients.

## Methods

### Literature Search Strategy

We have performed and reported this meta-analysis according to the Preferred Reporting Items for Systematic Reviews and Meta-Analyses (PRISMA) guidelines [11]. Relevant studies were identified and selected by searching the databases–Medline, PubMed, EMBASE, Cochrane databases and Google Scholar, from their date of inception to May 2014, using combinations of the search terms: “mediastinal lymph node dissection” OR “mediastinal lymph node excision” OR “lymphadenectomy” OR “mediastinal lymph node sampling” AND “non-small cell lung cancer” OR “NSCLC” AND “randomized controlled trial” OR “RCT”. There were no restriction of origin and languages. The reference lists of all retrieved articles were also reviewed and searched for further identification of potentially relevant studies. American Society of Clinical Oncology and the World Lung Cancer Conference were searched to identify unpublished studies. Each publication was carefully examined, including the names of authors, to avoid duplication of data. The supporting PRISMA checklist is available as supporting information (see Checklist S1).

### Selection Criteria

Studies were selected for inclusion in this analysis based on the following criteria: (1) Studies adopting randomized controlled trials to compare MLND vs. MLNS in early stage NSCLC patients; (2) No previous treatment for NSCLC; (3) Outcomes included overall survival, local recurrence, distant metastasis, and complications; (4) Studies were limited to human trials. Exclusion criteria for this analysis were as follows: (1) Case studies, review articles, and studies involving fewer than three patients; (2) Letters, editorials, and expert opinions without original data; (3) Studies lacking control groups; (4) Studies with no clearly reported outcomes of interest.

### Data Extraction and Quality Assessment

Two reviewers, XFH and JLJ, independently selected the trials and performed the data extraction according to a standard protocol. Any disagreements between the two reviewers were resolved by discussion or by consulting with the third reviewer (JMW). The collected data included several baseline characteristics: the first author or study group name, the year of publication, the number of patients enrolled, country of the population studied, the mean age, the interventions, the duration of follow-up, and the date of complications, distant metastasis, local recurrence, and overall survival. When data were missing or unclear in a paper, attempts were made to contact the authors for more information. The hazard ratios (HRs) of time-to-event data were directly extracted from the original studies or were estimated by reading off survival curves as suggested by Parmar et al. [12].

The quality of included RCTs was assessed using the tool of “risk of bias” according to the Cochrane Handbook (version 5.1.0) [13]. Sequence generation, allocation concealment, blinding, incomplete data and selective reporting were assessed, and each of them was graded as “yes(+)”, “no(–)” or “unclear(?)”, which reflected low risk of bias, high risk of bias and uncertain risk of bias, respectively. Two reviewers, XFH and QC, who were blinded regarding the source institution and the authors for each included RCTs independently assessed the methodologic quality. Disagreement between the two reviewers was settled by discussing with the third reviewer (JMW).

### Statistical Analysis

Statistical analysis was carried out using Review Manager 5.3 provided by The Cochrane Collaboration. Meta-analysis was performed using random-effect or fixed-effect methods, depending on the presence or absence of significant heterogeneity. Statistical heterogeneity between trials was evaluated by the χ^2^ and *I* ^2^ tests [14]. For the χ^2^ statistic, a *P* value<0.10 was considered statistically significant for heterogeneity; for the *I* ^2^ statistic, heterogeneity was interpreted as absent (*I* ^2^: 0%–25%), low (*I* ^2^: 25%–50%), moderate (*I* ^2^: 50%–75%), or high (*I* ^2^: 75%–100%) [15]. When heterogeneity was confirmed, the random-effect method was used. In the absence of statistically significant heterogeneity, the fixed-effect method was used to combine the results [16]. Time-to-event outcomes were compared using hazard ratio (HR). Dichotomous data were compared using risk ratio (RR) or odds ratio (OR). Respective 95% confidence intervals (CI) were calculated for each estimate and presented in forest plots. All statistical assessments were 2-sided, and a *P* value<0.05 was considered to indicate statistical significance.

## Results

### Search Results and Trial Characteristic

**Figure 1:**
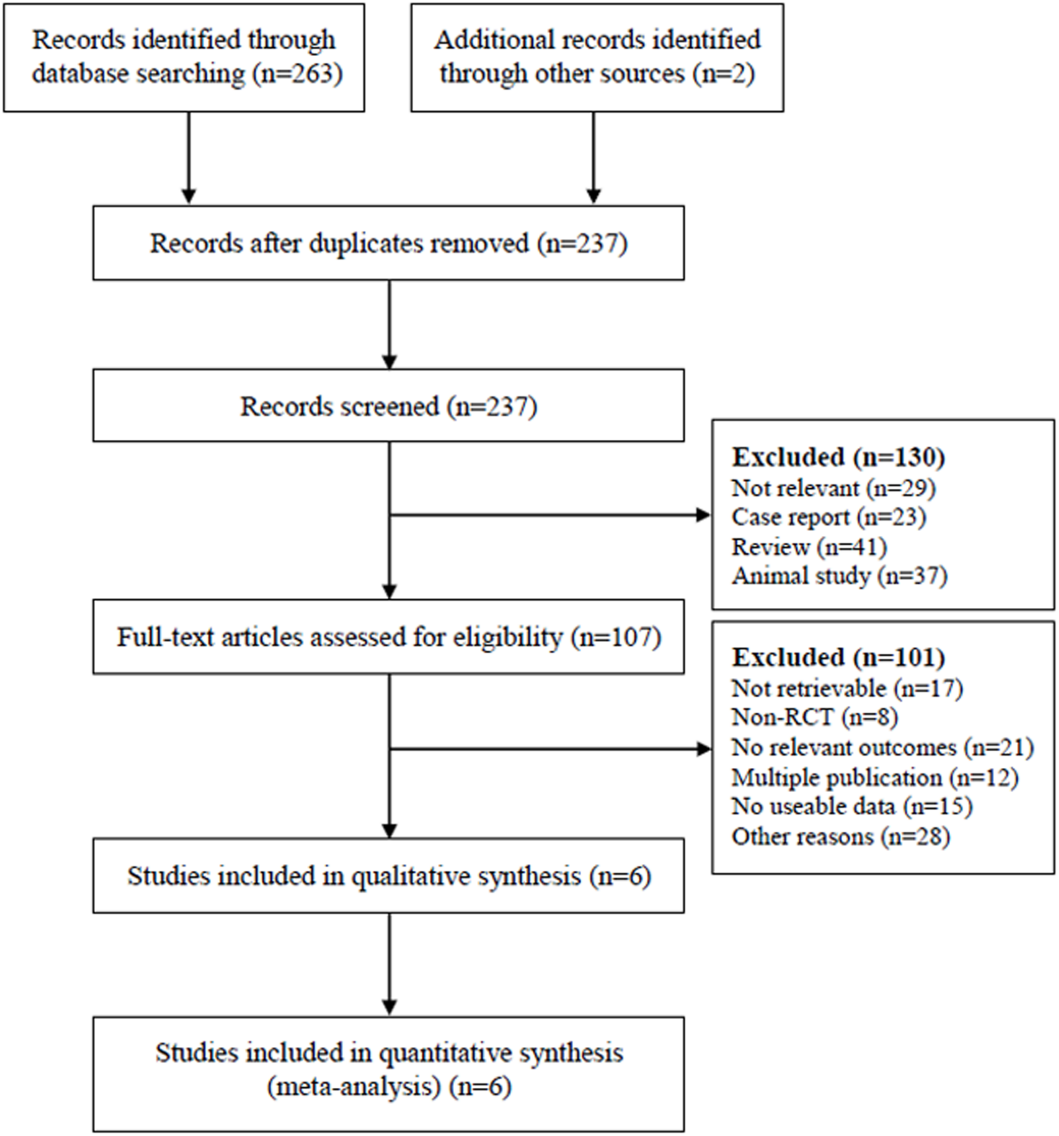
Flow diagram showing the selection process of articles. RCT, randomized controlled trial.

A total of 265 studies were identified by the searches. By scanning titles and abstracts, reviews, observational studies, case reports, and meeting abstracts were excluded. Therefore, 107 studies were included in the next round of review. After reading the full text of these articles, we removed 101 studies that did not meet the selection criteria. A diagram represents the flow of identification and inclusion of trials (Figure 1), as recommended by the PRISMA statement. As a result, six RCTs [10], [17]–[21] that included a total of 1791 patients were selected for meta-analysis; these, patients 906 (50.58%) had undergone MLND and 885 (49.42%) MLNS. Of the six included RCTs, two RCTs [10], [17] were studying the same patient population were conducted in America, two RCTs [18], [19] were studying the same patient population in Europe, and the remaining two RCTs [20], [21] in Asia. The details of the six RCTs were summarized in Table 1.

**Table 1:** Studies included in the meta-analysis.

First author,year, location	Participants	StudyGroup	Patients, n	Men, n	Age, y,median	Outcomes (MLND/MLNS)
Darling [10],2011, USA	N0 or N1NSCLC	MLND	525	272	67	Overall survival (52.4%/50.9%); local recurrence (5.7%/4.8%); distant metastasis (21.7%/22.3%)
		MLNS	498	257	68	
Allen [17],2006, USA	N0 or N1NSCLC	MLND	525	272	67	Complications (e.g., arrhythmia, prolonged air leakage, and pneumonia)
		MLNS	498	257	68	
Izbicki [18],1998, Germany	In stage I–IIIANSCLC	MLND	76	52	ND	Overall survival (70.6%/47.9%); local recurrence (28.9%/34.4%); distant metastasis (26.3%/31.2%)
		MLNS	93	73		
Izbicki [19],1994, Germany	In stage I–IIIANSCLC	MLND	82	56	58.5	Complications (e.g., arrhythmia, prolonged air leakage, and pneumonia)
		MLNS	100	80	60.9	
Sugi [20],1998, Japan	PeripheralNSCLC<2 cmdiameter	MLND	59	31	64.7±1.2	Overall survival (81.4%/83.9%); local recurrence (3.4%/3.6%); distant metastasis (10.2%/8.9%); complications (e.g., arrhythmia, prolonged air leakage, and pneumonia)
		MLNS	56	26	66.7±2.6	
Wu [21],2002, China	In stage I–IIIANSCLC	MLND	240	182	57	Overall survival (48.37%/36.98%); local recurrence (2.9%/4.8%); distant metastasis (22.5%/30.7%)
		MLNS	231	184	57	

**Abbreviations**: MLND, mediastinal lymph node dissection; MLNS, mediastinal lymph node sampling; NSCLC, non-small cell lung cancer; ND, not derived.

### Methodological Quality

**Figure 2:**
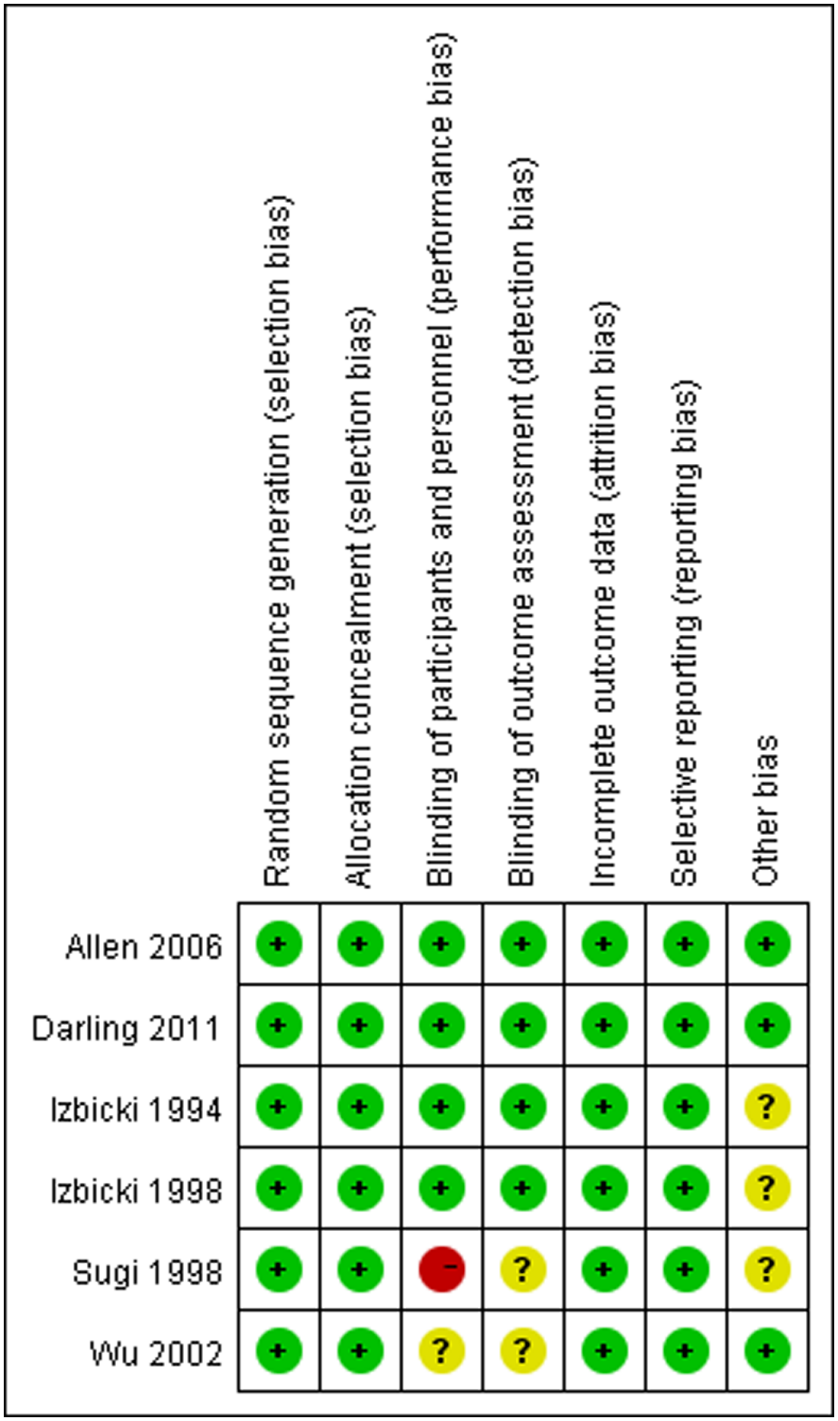
Risk of bias summary: review authors’ judgements about each methodological quality item for each included study. “+”, “−” or “?” reflected low risk of bias, high risk of bias and uncertain of bias respectively.

In the six included RCTs, methods of randomisation and allocation concealment were found to be adequate. Four RCTs [10], [17]–[19] were reported to be “double-blind”, other two RCTs [20], [21] were open-label studies. Two RCTs [10], [17] had conducted the intention to treat analysis. Figure 2 illustrates our opinion about each item of bias risk for included RCTs, most of the items were at “low risk” based on Cochrane handbook (version 5.1.0) [13], suggesting a reasonable good quality of RCTs.

### Overall Survival

**Figure 3:**
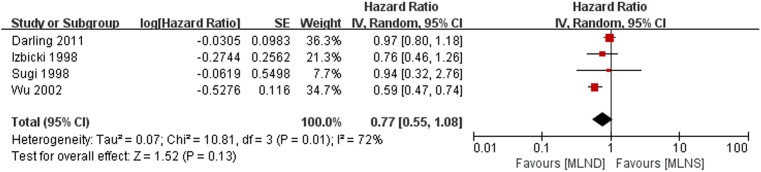
Forest plot of overall survival for the MLND vs. MLNS groups. MLND, mediastinal lymph node dissection; MLNS, mediastinal lymph node sampling; HR, hazard ratio; CI, confidence interval.

The meta-analysis results of overall survival are shown in Figure 3. Significant heterogeneity was detected between four RCTs [10], [18], [20], [21] being pooled (*P* = 0.01, *I* ^2^ = 72%). A random-effect model was therefore used for overall survival meta-analysis. The result, which showed there was no significant difference between MLND and MLNS groups with a pooled HR estimated at 0.77 (95% CI 0.55 to 1.08; *P* = 0.13).

### Local Recurrence

**Figure 4:**
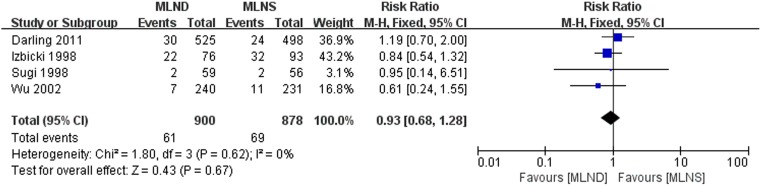
Forest plot of local recurrence for the MLND vs. MLNS groups. MLND, mediastinal lymph node dissection; MLNS, mediastinal lymph node sampling; RR, risk ratio; CI, confidence interval.

Figure 4 presents the forest plot of local recurrence rate. Four RCTs [10], [18], [20], [21] with complete data of local recurrence rates were included in the meta-analysis. No significant heterogeneity was detected between studies being pooled. A fixed-effect model was used for meta-analysis. The result with an RR = 0.93 (95% CI 0.68 to 1.28; *P* = 0.67) indicated no significant difference between MLND and MLNS groups.

### Distant Metastasis

**Figure 5:**
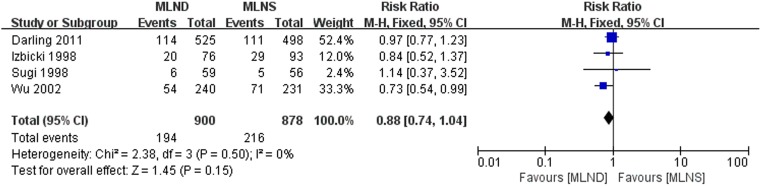
Forest plot of distant metastasis for the MLND vs. MLNS groups. MLND, mediastinal lymph node dissection; MLNS, mediastinal lymph node sampling; RR, risk ratio; CI, confidence interval.

The meta-analysis results of distant metastasis rate are shown in Figure 5. Four RCTs [10], [18], [20], [21] with complete data of distant metastasis rates were included in the meta-analysis. No significant heterogeneity was detected between studies being pooled. Thus fixed-effect model was selected. The result with an RR = 0.88 (95% CI 0.74 to 1.04; *P* = 0.15) indicated there was no significant difference between MLND and MLNS groups.

### Complications

**Figure 6:**
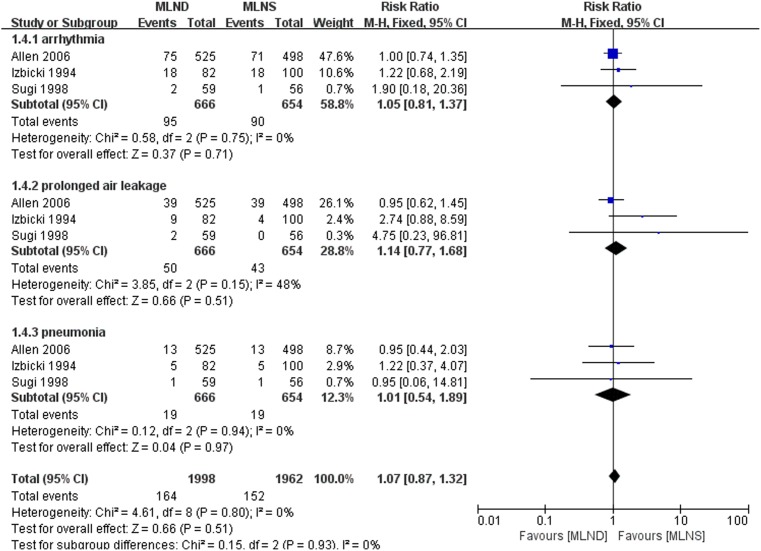
Forest plots of arrhythmia, prolonged air leakage, and pneumonia for the MLND vs. MLNS groups. MLND, mediastinal lymph node dissection; MLNS, mediastinal lymph node sampling; RR, risk ratio; CI, confidence interval.

Figure 6 presents the forest plots of complications including arrhythmia, prolonged air leakage, and pneumonia. Three RCTs [17], [19], [20] with complete data of these complications were included in the meta-analysis. No significant heterogeneity was detected between studies being pooled. Thus fixed-effect model was selected. The results of the meta-analyses indicated that MLND was associated with similar rates of arrhythmias (RR = 1.05, 95% CI 0.81 to 1.37; *P* = 0.71), prolonged air leakage (RR = 1.14, 95% CI 0.77 to 1.68; *P* = 0.51), and pneumonia (RR = 1.01, 95% CI 0.54 to 1.89; *P* = 0.97) compared to MLNS. There was no statistically significant difference in these specific complications between the two groups.

**Figure 7:**
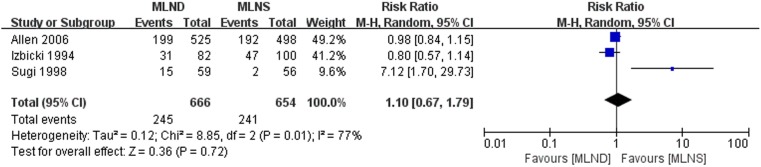
Forest plot of total complications for the MLND vs. MLNS groups. MLND, mediastinal lymph node dissection; MLNS, mediastinal lymph node sampling; RR, risk ratio; CI, confidence interval.

The meta-analysis results of total complications, which included neurological injury, arrhythmia, prolonged air leakage, pneumonia, empyema, chylothorax and bronchopleural fistulas, are shown in Figure 7. Significant heterogeneity was detected between three RCTs [17], [19], [20] being pooled (*P* = 0.01, *I* ^2^ = 77%). A random-effect model was used for total complications meta-analysis. The result, which showed there was no significant difference between MLND and MLNS groups with a pooled RR estimated at 1.10 (95% CI 0.67 to 1.79; *P* = 0.72).

## Discussion

Controversies still exists as to the need for MLND vs. MLNS for the cure of early stage NSCLC. This meta-analysis evaluated the overall survival, local recurrence, distant metastasis and complications of MLND vs. MLNS in early stage NSCLC patients using the best evidence available to date. We included results reported from six RCTs, with a total of 1,791 patients, of whom 906 (50.58%) underwent MLND and 885 (49.42%) underwent MLNS, included in the primary meta-analysis. Compared to MLNS in NSCLC patients, there was no statistically significant difference in MLND on overall survival. In addition, the results in our meta-analysis indicated that local recurrence rate, distant metastasis rate, and complications rate were similar, no significant difference found between the two groups.

Our meta-analysis showed that MLND was not associated with a statistically significant increase in overall survival compared with MLNS for the treatment of NSCLC patients (HR = 0.77, 95% CI 0.55 to 1.08; *P* = 0.13). Although the point estimates in the current meta-analysis indicate a slight benefit in MLND, these did not reach statistical significance. This result that is consistent with those of other studies. The ACOSOG Z0030 study [10] reported no difference in long-term survival between MLND and MLNS during pulmonary resection for patients with T1 or T2, N0 or nonhilar N1 NSCLC. Furthermore, both Izbicki et al. [18] and Sugi et al. [20] had reached similar conclusions. However, Wu et al. [21] pointed out in their prospective randomized trial that the MLND group showed significantly better survival compared with the MLNS group. In addition, a meta-analysis also concluded that MLND improve long term survival in stage I and IIIA NSCLC patients. In their pooled analysis of three RCTs [18], [20], [21] there was a significant reduction in the risk of death in the MLND group with a HR estimated at 0.78 (95% CI 0.65 to 0.93; *P* = 0.005) [8], [9]. In contrast with our study, their meta-analysis did not include the ACOSOG Z0030 trial, which is a multi-institutional prospective randomized trial.

However, these results in our meta-analysis of overall survival should be interpreted with caution because the heterogeneity of the data was high (*P* = 0.01, *I* ^2^ = 72%), and higher heterogeneity implies greater variation in true effect sizes as a consequence of various confounding factors. In the ACOSOG Z0030 trial [10], all patients had rigorous systematic node sampling prior to randomization, so that the proportion of patients with N2 disease was reduced. In addition, there was also has a lower proportion of N2 disease in Sugi’s study [20], which is a randomized trial conducted in patients with clinical stage I small (<2 cm) T1 NSCLC. Therefore, potential benefits of MLND might be minimized or negated in these two RCTs. Conversely, there was a higher proportion of patients with N2 disease in the remaining two RCTs [18], [21], which may result in a potential benefit to MLND. Our meta-analysis pooled all these four RCTs [10], [18], [20], [21], which may have markedly diluted any potential survival benefit seen in higher stage patients. Therefore, it could somehow make the results more scientific and credible in this meta-analysis.

Another source of heterogeneity is the methods of lymph node sampling leading to different rates of upstaging. When adequate lymph node sampling is not performed, the true N stage would remain unrecognized because all the lymph nodes are not dissected and pathologically examined, which may result in a spurious downstaging of such patients in MLNS groups. As a result, the so-called “Will Rogers” phenomenon, some potential benefits to MLND might be due at least in part to an imbalance within the groups with respect to the number of patients with lymph node involvement at multiple levels of the N2 region [19], [22], [23].

In Sugi’s study [20], the node positive N2 rate was similar in both groups, and the proportion was 12% and 14% of MLND and MLNS groups, respectively. Darling et al pointed out that there was only 4% of patients appeared to be upstaged to pN2 by complete dissection in their ACOSOG Z0030 trial [10] which had performed rigorous systematic node sampling prior to randomization. By contrast, the stage shift was more significant in the clinically staged “all-comer” trials of Izbicki et al. [18] and Wu et al. [21], and the rates of upstaging were higher than the other two RCTs [10], [20]. Izbicki et al. [18] reported that the number of pN2 levels in MLND group was 42% greater than that in MLNS group (*P* = 0.007). Because adjuvant chemotherapy is now the standard of care for patients with pN2, these increased node positive patients would result in additional survival at 5 years of 1–7% of participants as a result of appropriate administration of chemotherapy. Multiple studies have demonstrated that the clear benefit for adjuvant chemotherapy is in patients with node-positive NSCLC [24]–[30]. As there was no evidence or policy for adjuvant chemotherapy at the time of conduct of these trials, this study cannot evaluate the potential added survival benefit for patients upstaged by MLND compared to MLNS.

On the other hand, it is noteworthy that performing rigorous systematic node sampling may lead to more node treatment than the current standard of care [31]. Therefore, it is plausible that patients in these two RCTs (ACOSOG Z0030 trial [10] and Sugi et al. [20]) received better treatment in MLNS groups than is usually the case outside of a clinical trial.

In terms of local recurrence and distant metastasis, whether MLND might decrease the incidence of local recurrence and distant metastasis after complete resection for NSCLC is still a question that remains unanswered. In our meta-analysis, no significant differences were observed in local recurrence and distant metastasis between MLND and MLNS. Similarly, in the ACOSOG Z0030 trial [10], the authors found MLND does not affect the rate of local recurrence or distant metastasis. Izbicki et al. [18] indicated that recurrences rates tended to be reduced among patients who underwent MLND, but the decreases were not statistically significant. A randomized trial comparing MLND and MLNS in patients with clinical stage I small (<2 cm) T1 NSCLC (87% non-squamous cancers), Sugi et al. [20] reported there were two local and six distant recurrences in the MLND group (10%) and two local and five distant recurrences in the MLNS group (13%), no significant difference in the recurrence rate was found between the two groups.

The complication of MLND vs. MLNS in NSCLC patients is an intriguing question. Some arguments [32] against MLND include causing more complications, prolonging the hospitalization and increasing mortality, one potential explanation may be that MLND requires a more extensive mediastinal dissection. However, our meta-analysis showed that there was no statistically significant difference in complications between MLND and MLNS. These results of our meta-analysis are generally in agreement with the ACOSOG Z0030 trial [17]. In this trial, Allen et al. reported the total complications rate was 37.9% for MLND and 38.6% for MLNS, no statistically significant difference in any specific complication between the two groups. Furthermore, Izbicki et al. [19] observed a longer operation time caused by MLND, but the rate of complications was not influenced by the type of MLND. Additionally, they found the 30-day mortality was not statistically different between patients with MLND or MLNS.

Some limitations in this meta-analysis need to be acknowledged. Firstly, publication bias could not be excluded. Not only because of the positive outcome studies may be preferentially published [33]–[35], but it could also result from reporting negative studies of lymphadenectomy to justify the very common practice of not removing any lymph nodes. Secondly, a potential for a selection bias exists since the studies included in our meta-analysis did not include information published in textbooks and abstract only publications. Thirdly, the quality of the included studies may influence the power of our meta-analysis. Two RCTs [20], [21] were open-label studies could somehow reflect a detection bias in the rates of local recurrence, distant metastasis and complications, except for overall survival as it is a hard end point. Fourthly, this meta-analysis might be dominated by the ACOSOG Z0030 trial [10], [17], which is the largest RCT among the included RCTs. What's more, other factors, such as different ethnic mix, different therapy strategies, different lengths of follow-up, and different proportions lost to follow-up may confer limitations on this meta-analysis.

In conclusion, the results of our meta-analysis indicated that there was no statistically significant difference in overall survival, local recurrence, and distant metastasis between MLND and MLNS in early stage NSCLC patients. Furthermore, no evidence was found that MLND increased complications compared with MLNS. However, due to significant staging heterogeneity between RCTs, whether or not MLND is superior to MLNS for stage II–IIIA remains to be determined.

## Supporting Information

Checklist S1 **PRISMA Checklist.**
(DOC)
